# Classification of Overt and Covert Speech for Near-Infrared Spectroscopy-Based Brain Computer Interface

**DOI:** 10.3390/s18092989

**Published:** 2018-09-07

**Authors:** Ernest Nlandu Kamavuako, Usman Ayub Sheikh, Syed Omer Gilani, Mohsin Jamil, Imran Khan Niazi

**Affiliations:** 1 Centre for Robotics Research, Department of Informatics, King’s College London, London WC2B 4BG, UK; 2 Basque Center on Cognition, Brain and Language, 20009 Donostia, Spain; u.sheikh@bcbl.eu; 3 Department of Robotics and Artificial Intelligence, National University of Sciences and Technology, Islamabad 24090, Pakistan; omer@smme.nust.edu.pk (S.O.G.); mohsin@smme.nust.edu.pk (M.J.); 4 Department of Electrical Engineering, Faculty of Engineering, Islamic University Medina, Al Jamiah 42351, Saudi Arabia; 5 Center for Chiropractic Research, New Zealand College of Chiropractic, Auckland 1010, New Zealand; imran.niazi@nzchiro.co.nz; 6 SMI, Department of Health Science and Technology, Aalborg University, 9100 Aalborg, Denmark; 7 Health and Rehabilitation Research Institute, AUT University, Auckland 1010, New Zealand

**Keywords:** brain computer interface, near infrared spectroscopy, overt and covert speech, unsupervised feature extraction, Broca’s area, decoding speech

## Abstract

People suffering from neuromuscular disorders such as locked-in syndrome (LIS) are left in a paralyzed state with preserved awareness and cognition. In this study, it was hypothesized that changes in local hemodynamic activity, due to the activation of Broca’s area during overt/covert speech, can be harnessed to create an intuitive Brain Computer Interface based on Near-Infrared Spectroscopy (NIRS). A 12-channel square template was used to cover inferior frontal gyrus and changes in hemoglobin concentration corresponding to six aloud (overtly) and six silently (covertly) spoken words were collected from eight healthy participants. An unsupervised feature extraction algorithm was implemented with an optimized support vector machine for classification. For all participants, when considering overt and covert classes regardless of words, classification accuracy of 92.88 ± 18.49% was achieved with oxy-hemoglobin (O2Hb) and 95.14 ± 5.39% with deoxy-hemoglobin (HHb) as a chromophore. For a six-active-class problem of overtly spoken words, 88.19 ± 7.12% accuracy was achieved for O2Hb and 78.82 ± 15.76% for HHb. Similarly, for a six-active-class classification of covertly spoken words, 79.17 ± 14.30% accuracy was achieved with O2Hb and 86.81 ± 9.90% with HHb as an absorber. These results indicate that a control paradigm based on covert speech can be reliably implemented into future Brain–Computer Interfaces (BCIs) based on NIRS.

## 1. Introduction

Locked-in syndrome (LIS) is a neuromuscular disorder described as near-complete paralysis with preserved awareness and cognition [1]. Patients with LIS are left with very few degrees of freedom ranging from restricted eye movement (classic LIS) to complete immobility (total LIS) [2]. The most common cause of LIS is a stroke or a traumatic brain injury (31%) or a cerebrovascular disease (52%) [3]. According to Blain et al. [4], more than half a million people worldwide are affected by LIS. Once an LIS patient has become medically stable, his/her life span can be significantly prolonged. With very minor chances of motor recovery and poor quality of life, healthy individuals and medical experts often find themselves wondering if such a life is worth fighting for [5]. Recent advances in Brain–Computer Interfaces (BCI) can potentially redefine the quality of life (QoL) of such patients by providing them with muscle independent communication channel to communicate and interact with their environment. When considering a BCI for a speech-deprived patient, one must also take into account the nature of the patient’s impairment. Interfaces that rely on movement of non-vocalized articulators or minimal control of muscles for restoration of communication are not feasible for those suffering from LIS. Non-invasive BCIs currently available for such patients are mostly limited to those based on Electroencephalographic (EEG) signals with many challenges [6]. An alternative technique based on Near-Infrared Spectroscopy (NIRS) may be used to create BCIs with potentially less challenges due to the non-electric properties of the response.

Near-Infrared Spectroscopy (NIRS), a relatively recent non-invasive neuroimaging technique [7,8], makes use of electromagnetic radiation in near-infrared region (650–900 nm) [9] in order to measure functional activation in cortical areas 1–3 cm beneath the scalp [10]. Among dominant chromophores that also happen to be biologically relevant markers for brain function are oxy-hemoglobin (O2Hb) and deoxy-hemoglobin (HHb) [11]. NIRS has emerged during the last decade as a promising non-invasive neuroimaging technique and has been used to map different areas of the brain including primary motor cortex [12], visual [13], as well as cognitive and language functional areas [8,14,15,16,17,18,19,20,21]. Naito et al. [22] presents a study conducted on 40 Amyotrophic Lateral Sclerosis (ALS) patients including 17 in locked-in state in order to investigate the use of high-level cognitive tasks as a control signal for a BCI. Single-channel measurements were recorded over the prefrontal cortex while participants performed mental tasks corresponding to ‘yes’/‘no’ in response to a series of questions. Instantaneous amplitude and phase of the NIRS signal were selected as features and a non-linear discriminant classifier was used to achieve an average classification accuracy of 80% (for 23 out of 40 participants). While EEG-BCIs haven’t succeeded in breaking the silence of Completely Locked-in (CLI) patients [23], a recent clinical study [24] presents a Class IV case evidence proving that NIRS-BCIs could significantly improve the QoL of such people by allowing them to regain basic communication. Nevertheless, studies focusing on directional actions, which might be beneficial for moving in space, are lacking.

In this study, we worked out the feasibility of detecting user’s intention by decoding his/her speech. We reasoned that if a small set of overtly/covertly said words can reliably generate hemodynamic activity discernible with NIRS, then a control paradigm based on speech may be implemented into future NIRS-BCIs. Therefore, the aim was to investigate if an intuitive BCI based on NIRS can be created that may ultimately pave the way for the rehabilitation of patients suffering from neuromuscular disorders such as LIS for improved QoL.

## 2. Materials and Methods

Among several choices related to signal acquisition for speech classification that were made during this study, two aspects are worth mentioning: (1) Broca’s area (speech center) was chosen as it plays an important role in speech processing [25] and as activation within this area is unavoidable during speech [26]; (2) Use of six directional words i.e., Up, Down, Right, Left, Forward, and Backward, since a navigational approach allows an intuitive control and also it is commonly used in many BCI applications (e.g., selection of letters on a virtual keyboard [27] or navigation of a mouse [28] or a wheelchair [29]).

### 2.1. Participants

Since the issue of language representation in bilinguals is still a topic of debate [30], it was chosen to include only those participants that are monolingual speakers. Once the experiment was approved by the “Biomedical and Scientific Research Ethics Committee of National University of Sciences and Technology”, a total of eight healthy participants, aged between 23 and 29, were selected for the study. All of them gave written informed consent in accordance with the Declaration of Helsinki. As hemispheric language lateralization varies significantly among left-handed people, a quantitative measure known as Edinburgh Inventory Test [31] was used to assess the handedness of the participants. The analysis indicated five of the participants to be right-handed with Laterality Quotient (LQ) > 70, two to be both-handed (LQ = 10 and 30), and one to be left-handed (LQ = −58).

### 2.2. Data Acquisition

**Figure 1:**
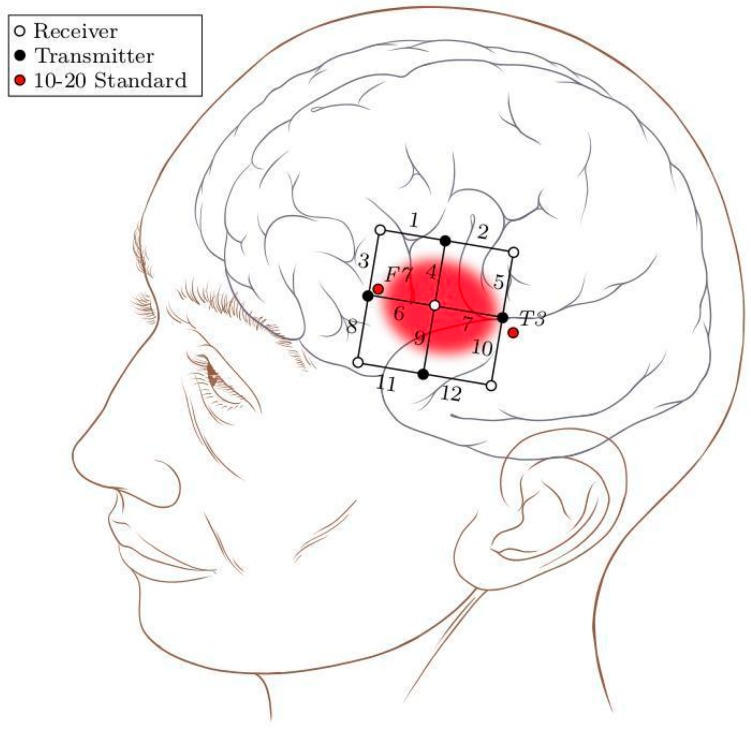
A 12-channel square patch with an inter-optode distance of 3 cm was placed with T3 on one end and F7 on the other. An estimation of the underlying anatomical structures is also shown with Broca’s area highlighted. The head diagram is available at [35].

The experiment was conducted in a dimly lit room, with lights switched off and curtains closed. In order to minimize interference due to low frequent Mayer waves (~0.1 Hz) [32], participants were comfortably seated in a tilted position (40° from upright) with their eyes closed. When prompted to open their eyes, they were able to see the screen. Participants were instructed not to move throughout the experiment as this might produce motion artifacts and thus undesirably introduce noise in the signal. A multi-channel Oxymon device with associated software Oxysoft (Artinis Medical Systems, Gelderland, The Netherlands) was used for measurement of NIRS. A 12-channel square template (6 × 6 cm^2^) with an inter-optode distance of 3 cm was used to cover left inferior frontal gyrus (IFG). To locate in a specific Broca’s area, the international 10–20 standard for EEG was used and the template was placed with T3 on one end and F7 on the other (see Figure 1). This is done in accordance with [33] which has shown F7 to cover anterior portion of pars triangularis and T3 to be posterior to IFG. The area of the template was inspired by [34], which has shown the size of Broca’s area to be below 6 cm. NIRS signals were acquired with a sampling frequency of 10 Hz.

### 2.3. Experimental Procedure

**Figure 2:**
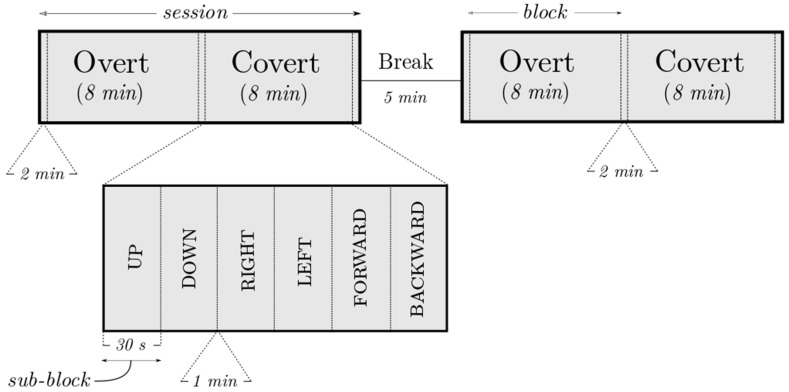
An example of the experiment layout showing the two sessions, overt/covert blocks (8 min) alternating with the relaxation periods (2 min) and a zoomed-in view of the sub-blocks (30 s; 30 repetitions of a word). The figure is not drawn to scale.

The experiment (see Figure 2) was composed of two sessions with a 5 min break between them so as to collect as many trials as possible while allowing some time for relaxation too. Each session was composed of a selected set of directional words (up, down, right, left, forward, and backward) overtly and covertly said for 30 s, at a frequency of 1 Hz. Within a session, the order of blocks (overt, covert) as well as sub-blocks (‘UP’, ‘DOWN’) was randomized. A 1 min break was allowed between sub-blocks to avoid any carry-over effect; similarly, an additional pause of one minute was also added before each block. Each session thus lasted for about 22 min and a complete experiment had a duration of about 49 min excluding mounting time.

The participants were guided through the experiment by a low-frequency sound (300 Hz, 0.5 s in duration) that instructed them to open their eyes, read the word and either overtly or covertly say the word. The sound was played at a low volume to avoid shocking the participants. All textual stimuli including words for overt and covert tasks were presented in white against black background. The screen also had a 1 Hz counter/indicator to guide the participants to maintain a constant pace of one word per second. After each sub-block (30 s interval, each word spoken 30 times), the screen would go back to signal a break and participants were instructed to close their eyes and rest. The overt–covert switch and the termination of the session were indicated on the screen by stating overt/covert and block respectively after one minute of black screen. The procedure based on such audio-visual instruction was chosen so as to minimize the activity in the IFG and its surroundings by maximizing the use of the visual cortex and minimizing the use of the auditory cortex—close to T3. Similarly, during covert blocks, it was ensured that the participant does not make mouth movement as this might induce additional changes in cerebral hemodynamics [36] and thus disturb the response associated with a particular activation trial. Prior to the experiment, it was also confirmed that the participant could easily hear the sound and is familiar with the task and the stimulus.

### 2.4. Data Analysis

**Figure 3:**
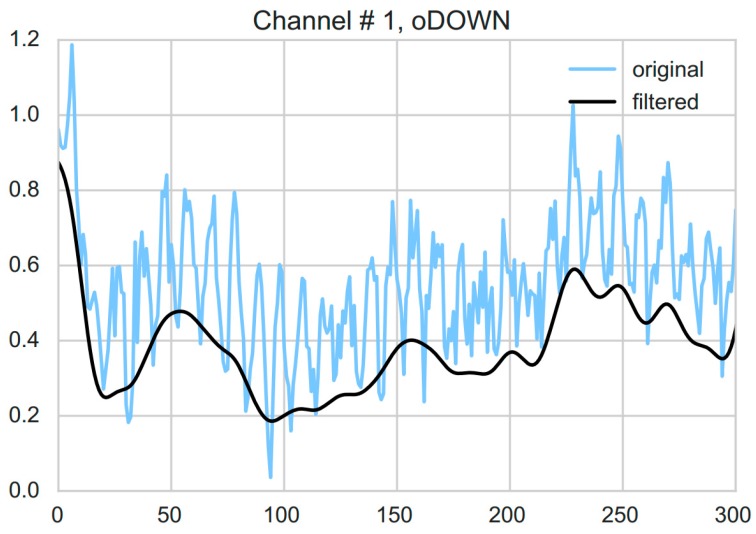
An example showing both raw (blue) and filtered (black) HHb response corresponding to the first channel of one of the sub-blocks i.e., overt DOWN of one of the subjects. Filtering was performed by first removing the linear trends from the response and then using a fourth order Butterworth filter with a cut-off frequency of 0.5 Hz to account for the effects of the cardiac pulse.

Preprocessing involved linear detrending followed by low-pass filtering as reported in an earlier study by Coyle [32]. Specifically, first, a filter was applied to remove the linear trends in the metabolic response by minimizing the least-squares error and subsequently subtracting the linearly increasing baseline. Second, a fourth order Butterworth filter with a cut-off frequency of 0.5 Hz was used to account for the effects of the cardiac pulse (see Figure 3). Forward and reverse filtering was applied so as to prevent phase distortion. Before any further analysis, all rest events including the start of session rests, between session breaks and between sub-blocks rests were removed from the data.

Prior to feature extraction and classification, the data was transformed into examples. Recall that NIRS signals corresponding to each spoken word were recorded as ten data points (sampling frequency = 10 Hz; see Section 2.2) in a 12-dimensional space (12 channels). To convert each of these 10 × 12 matrices into an example, the rows were horizontally stacked to create a 120-dimensional feature vector. This created a total of 30 examples per each sub-block.

Scikit-learn library [37] was used for feature extraction and classification analysis. To start with, each feature was individually scaled to a range between 0 and 1. To reduce the dimensionality of the data, and thus reduce the chances of overfitting, principle component analysis (PCA), with all parameters set to default values, was used. Since none of the components was excluded, it was a loss-less change of the coordinate system to a subspace spanned by the samples/examples. For classification, Support Vector Machine (SVM), with radial basis function as kernel, was used. To simulate a pseudo-online BCI, the unshuffled trials of each class were split into two parts, first 80% of the trials were used for training while last 20% were used for testing. To search for the optimal C and γ hyper parameters of the SVM, two-dimensional grid search using nested 3-fold cross-validation was implemented.

Two types of classification analysis were performed i.e., general classification and pair-wise classification. In general classification, three different classification strategies were considered: (1) binary classification of overt and covert (DiffOvCov); (2) multi-class classification of overt words (SepOv) and (3) multi-class classification of covert words (SepCov). Since DiffOvCov was a binary classification problem, the chance-level performance was 50%; for SepOv and SepCov six-class problems, the chance-level was at 16.67%. For multi-class classification, one vs. rest (or one vs. all) method was used. In pairwise classification, the analysis was performed while concentrating on a pair of words within overt and covert classes e.g., overt UP vs. overt DOWN. Considering all possible combinations, a total of fifteen pairs and thus fifteen binary classification problems were created for each of overt and covert classes. The purpose of this analysis was to investigate which pairs of words are most differentiable based on patterns of activity and thus show highest classification performance.

To determine whether the classification accuracy is statistically significantly different from chance-level, one-sample *t*-test was performed. The statistical significance was established by comparing the probability (*p*-value) under the null hypothesis with the significance level of 0.05. For comparison of the performance between HHb and O2Hb for each of the classification strategies (DiffOvCov, SepOv, and SepCov), paired t-test was used. The same was also used to compare between the performance corresponding to overt and covert words.

## 3. Results

**Figure 4:**
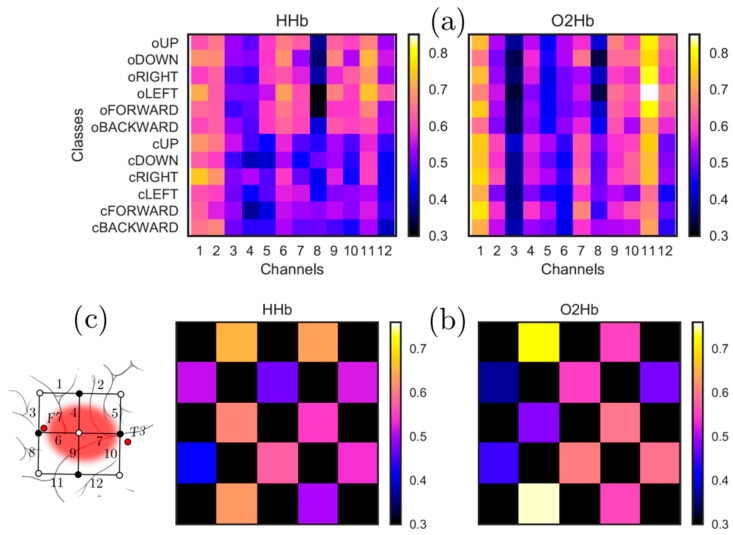
(**a**) shows mean activation for each of the words vs. channels for both O2Hb and HHb, and it can be seen that the channel providing the maximum value is channel # 1 for HHb and channel # 11 for O2Hb (**b**) shows the mean activity as a topographical map with arrangement of channels same as the 12-channel patch as shown in (**c**) (also see Figure 1). The black areas in (**b**) are the parts of the patch not covered by any sensor.

Considering mean activation across all participants, the channel providing the maximum value was channel # 1 for HHb and channel # 11 for O2Hb (see Figure 4).

### 3.1. General Classification

**Figure 5:**
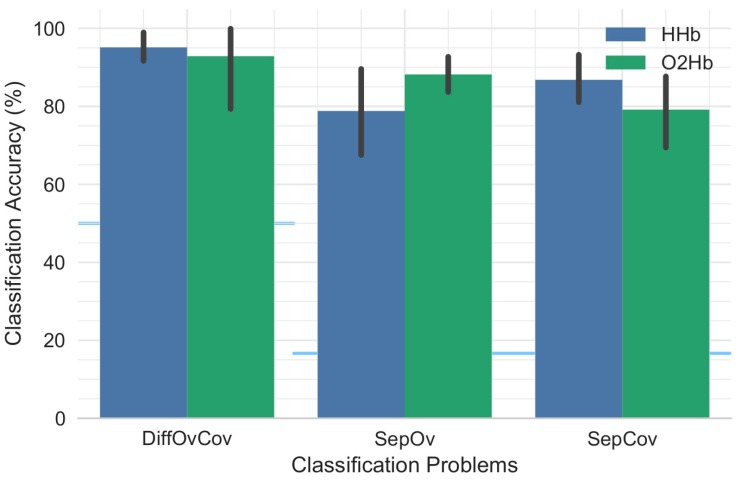
Mean classification accuracy for the different classification strategies. The height of each bar indicates mean classification performance and the error bars indicate bootstrapped 95% confidence intervals estimated by resampling participants (1000 bootstrap samples). The blue horizontal lines show the chance-level performance for the corresponding pairs of bars.

The overall classification performance across participants is provided in Figure 5. Table 1 provides detailed results for each individual participant showing that performance is participant dependent. No significant differences were found between classification performance for HHb and O2Hb with respect to DiffOvCov (*t*(8) = 0.40, *p* = 0.70), SepOv (*t*(8) = −1.70, *p* = 0.13), and SepCov (*t*(8) = 1.90, *p* = 0.10). Similarly, classification performance for covert speech was not significantly different from overt speech (*t*(8) = −1.56, *p* = 0.16 for HHb; *t*(8) = 1.42, *p* = 0.20 for O2Hb).

### 3.2. Pairwise Classification

Classifying pairs of active classes revealed that the most separable pair is dependent on the speech modality (covert vs. overt) and signal modality (HHb vs. O2Hb). Using O2Hb, the pairs with the highest accuracy were <Right, Left: 99.48 ± 1.47%> and <Left, Backward: 98.96 ± 2.95%> for overt and covert speech, respectively. When using HHb, the pairs with the highest accuracy were <Up, Down: 99.48 ± 1.47%> and <Left, Forward: 99.48 ± 1.47%> for overt and covert speech, respectively.

## 4. Discussion

This study focused on the classification accuracies in order to assess the feasibility of a NIRS based BCI with speech as a control source. To our best knowledge, exploiting the advantages of NIRS for identification of multiple distinct outcomes from speech on a relatively localized area—Broca’s area—as a control scheme for a BCI forms a novel approach. In general, no statistically significant differences were observed in the classification outcomes for HHb and O2Hb, which emphasizes that the choice of an absorber is not of great importance. The average classification accuracy for each of the combination of classes was comparable to those achieved by Holper and Wolf [38] in similar studies. They used an LDA classifier to discriminate between motor imagery (MI) simple and complex finger tapping using a 3-channel template around F3 on 12 participants while we used SVM to classify overt and covert speech events using a 12-channel template around T3 and F7 on 8 participants. They used hand-crafted features such as mean, variance and skewness while we used unsupervised PCA-based feature reduction. They only used a single best performing channel for feature extraction and classification while we used all 12 channels.

Although the laterality of Broca’s area can vary, especially for left handers, as shown in an MRI study by Keller et al. [34], it could not be observed in this study. One reason for relatively low classification accuracies in SepOv and SepCov might be the length of the experiment. Even though it was kept as short as possible, it was still longer than the time any participant could refrain from using their inner voice and consequently unintentionally produce covert speech. Moreover, sitting in a dark room with no lights lit and their eyes closed might leave the participants in a daydreaming state which was also reported as a source of problem in a similar study by Herrmann et al. [39].

Detailed investigation in the form of pair-wise comparisons based on classification accuracies allowed us to find best pairs of overtly/covertly spoken words for each of the participants and absorbers. The most separable pair among all overt and covert events turned out to be covert <Right, Left> with O2Hb as an absorber. However, with HHb, the overt <Up, Down> pair was found to be as discriminable as covert <Left, Forward>. This is an important finding because many studies [38,40] tend to use O2Hb due to its high amplitude response. This shows that the size of the response does not necessarily mean greater discriminative capability.

### Limitations

One important limitation of this study is its inclusion of healthy participants while the target users are patients with neuromuscular disorders and thus, the results cannot be generalized to patients. It is very sensible to expect a drop in performance but this decline will also depend on a number of other factors including severity of impairment, experimental design, and NIRS classification pipeline. Also, normal skin blood flow under the electrodes could have contributed differently towards the performance, this need to be taken in to future studies. Another limitation that needs to be overcome before moving on to a real-world implementation has to do with the processing of signals. Whereas the proposed classification pipeline turned out to be very important in assessing the feasibility of a novel BCI, the processing was done offline. Keeping in view however, the simplicity of the proposed feature extraction and classification pipeline, it is expected that online implementation will be equally feasible.

## 5. Conclusions

This study investigates an intuitive BCI paradigm based on NIRS and demonstrates that speech can be harnessed as an intuitive control source for a NIRS-BCI. The study also demonstrates that Broca’s area can be used to differentiate between overt and covert speech regardless of words with binary classification accuracy up to 95%, between six overtly spoken words with classification accuracy up to 88%, and between six covertly spoken words with classification accuracy up to 87%.

## Figures and Tables

**Table 1:** Individual classification performance sorted by handedness of the participants. The first number is classification accuracy based on HHb and the second is based on O2Hb.

Participant	Handedness	DiffOvCov	SepCov	SepOv
1	Right	91.67/100.00	87.50/86.11	69.44/91.67
2	Right	89.58/96.53	76.39/75.00	72.22/81.94
3	Right	100.00/100.00	98.61/69.44	87.50/86.11
4	Right	100.00/99.31	75.00/54.17	97.22/100.00
5	Right	87.50/47.22	76.39/75.00	52.78/88.89
6	Both	100.00/100.00	87.50/80.56	66.67/84.72
7	Both	100.00/100.00	95.83/93.06	91.67/77.78
8	Left	92.36/100.00	97.22/100.00	93.06/94.44
mean		95.14/92.88	86.81/79.17	78.82/88.19
*t*(8)		23.68/6.56	20.03/12.36	11.15/28.41
*p*-value		<0.001	<0.001	<0.001
